# Switching of vascular cells towards atherogenesis, and other factors contributing to atherosclerosis: a systematic review

**DOI:** 10.1186/s12959-020-00240-z

**Published:** 2020-10-28

**Authors:** Ovais Shafi

**Affiliations:** Sindh Medical College - Dow University of Health Sciences, Karachi, Pakistan

**Keywords:** Atherogenesis, Atherosclerosis, Ageing, Changes in vasculature, Vascular homeostasis, Signaling pathways, Vascular microenvironment, Gene expression, Inflammation, Oscillatory blood flow

## Abstract

**Background:**

Onset, development and progression of atherosclerosis are complex multistep processes. Many aspects of atherogenesis are not yet properly known. This study investigates the changes in vasculature that contribute to switching of vascular cells towards atherogenesis, focusing mainly on ageing.

**Methods:**

Databases including PubMed, MEDLINE and Google Scholar were searched for published articles without any date restrictions, involving atherogenesis, vascular homeostasis, aging, gene expression, signaling pathways, angiogenesis, vascular development, vascular cell differentiation and maintenance, vascular stem cells, endothelial and vascular smooth muscle cells.

**Results:**

Atherogenesis is a complex multistep process that unfolds in a sequence. It is caused by alterations in: epigenetics and genetics, signaling pathways, cell circuitry, genome stability, heterotypic interactions between multiple cell types and pathologic alterations in vascular microenvironment. Such alterations involve pathological changes in: Shh, Wnt, NOTCH signaling pathways, TGF beta, VEGF, FGF, IGF 1, HGF, AKT/PI3K/ mTOR pathways, EGF, FOXO, CREB, PTEN, several apoptotic pathways, ET – 1, NF-κB, TNF alpha, angiopoietin, EGFR, Bcl − 2, NGF, BDNF, neurotrophins, growth factors, several signaling proteins, MAPK, IFN, TFs, NOs, serum cholesterol, LDL, ephrin, its receptor pathway, HoxA5, Klf3, Klf4, BMPs, TGFs and others.

This disruption in vascular homeostasis at cellular, genetic and epigenetic level is involved in switching of the vascular cells towards atherogenesis. All these factors working in pathologic manner, contribute to the development and progression of atherosclerosis.

**Conclusion:**

The development of atherosclerosis involves the switching of gene expression towards pro-atherogenic genes. This happens because of pathologic alterations in vascular homeostasis. When pathologic alterations in epigenetics, genetics, regulatory genes, microenvironment and vascular cell biology accumulate beyond a specific threshold, then the disease begins to express itself phenotypically. The process of biological ageing is one of the most significant factors in this aspect as it is also involved in the decline in homeostasis, maintenance and integrity.

The process of atherogenesis unfolds sequentially (step by step) in an interconnected loop of pathologic changes in vascular biology. Such changes are involved in ‘switching’ of vascular cells towards atherosclerosis.

## Study design

The precise nature of onset, development and progression of atherosclerosis is not yet properly known. The role of ageing in atherosclerosis is very crucial. This study is concerned mainly with investigating the changes that occur in vasculature and are involved in ‘switching of vascular cells towards atherogenesis’. It also investigates other factors in relation to development of atherosclerosis including ageing, maintenance of vascular homeostasis, signaling pathways, gene expression, angiogenesis, vascular development, vascular cell differentiation and maintenance, vascular stem cells, endothelial, smooth muscle cells and others.

**Fig. 1:**
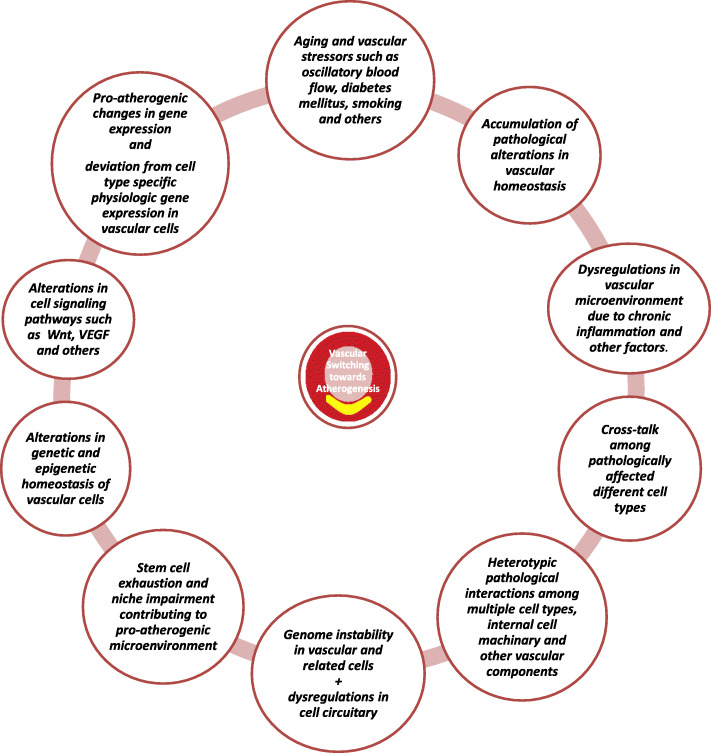
Illustration of major changes in vasculature responsible for ‘Vascular Switching towards ATHEROGENESIS’. The development of atherosclerosis is a complex multi-step process. This process unfolds in a step by step manner that switches the gene expression towards pro-atherogenic genes. This happens in an interconnected loop of pathological alterations in vasculature that lead to the disease development.

This research study finds evidence from already published research literature to find the proatherogenic changes in vasculature that lead or contribute to development of atherosclerosis (Fig. 1).

These are the ‘other factors’ implied by the title of the study, as their contributions to the development of atherosclerosis are explored in this study. They are also the limitations of this study.

**Fig. 2:**
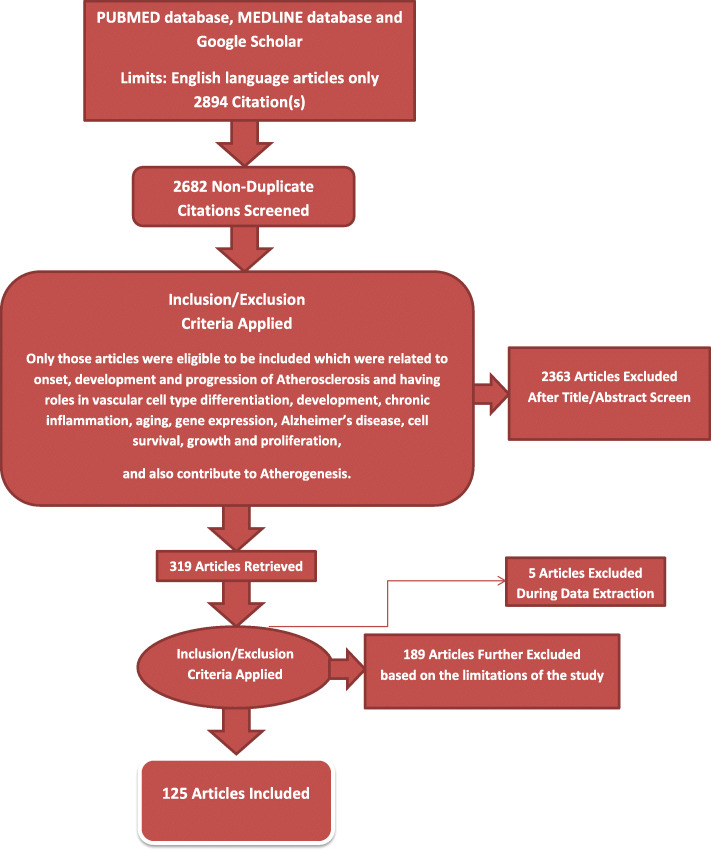
PRISMA Flow Diagram. This figure only highlights the methodology of the study in relation to its limitations. The limitations are detailed in the ‘methodology’ and in the beginning of ‘results’ sections. This figure represents graphically the flow of citations reviewed during the study

The limitations are further explained in ‘Methodology’, in the beginning of ‘Results’ section, and in other respective sections and headings (Fig. 2).

## Background

Atherosclerosis is a disease of elastic arteries, large and medium sized muscular arteries. It mostly occurs in abdominal aorta, coronary artery, popliteal artery and carotid artery. Risk factors involved include smoking, hypertension, hyperlipidemia, diabetes, but these factors are modifiable. Turbulent blood flow also plays a major role in switching the vasculature towards pro-atherogenic state. Age, gender and family history are non-modifiable risk factors. With increasing age, the role of all these risk factors becomes more and more severe in the development of atherosclerosis. One of the main goals of this study is to explore the role of ageing in switching vascular cells towards atherogenesis. It is important to note that not every elderly develops clinically significant atherosclerosis because many factors including genetic, environmental and behavioral play role in the disease development [1].

Aging increases the risk of atherosclerosis exponentially, the mechanisms via which aging contributes to the development of atherosclerosis are not yet properly determined [2]. Accumulation of DNA damage in endothelial cells, macrophages and vascular smooth muscle cells contribute to the ageing of vasculature and in turn contributes to the development of atherosclerosis [3, 4]. But this is just one of the many cascades of mechanisms that lead to the onset and development of atherosclerosis.

The exact etiology of atherosclerosis is not yet properly known. The etiological nature of endothelial dysfunction and changes in VSMCs gene expression that trigger neointimal proliferation is still not yet properly known. The task of this research work is to investigate the pathological changes in the vasculature that result in atherogenesis and contribute to the development of atherosclerosis. In the light of scientific literature, this study also suggests some hypothesis and predictions.

## Methods

PUBMED database, MEDLINE database, Google Scholar and online journals such as Circulation, BMC, Cardiovascular Research, PLOS Biology and others were searched with no date restrictions for published articles in relation to ‘atherosclerosis’.

Keywords used: atherogenesis, vascular homeostasis, aging, gene expression, signaling pathways, angiogenesis, vascular development, differentiation, maintenance, vascular stem cells, endothelial, smooth muscle cells**,** Shh, Wnt, NOTCH signaling pathways, TGF beta, VEGF, FGF, IGF 1, HGF, AKT/PI3K/ mTOR pathways, EGF, FOXO, CREB, PTEN, several apoptotic pathways, ET – 1, NF-κB, TNF alpha, angiopoietin, EGFR, Bcl − 2, NGF, BDNF, neurotrophins, growth factors, several signaling proteins, MAPK, IFN, TFs, NOs, serum cholesterol, LDL, ephrin and its receptor pathway, HoxA5, Klf3, Klf4, BMPs, TGFs and others

All these keywords were searched with terms such as atherogenesisAtherosclerosisSignaling pathways and those mentioned in eligibility criteria below such as “Aging and Atherosclerosis”, “Wnt signaling and Atherogenesis” and others. This was the main search strategy throughout the study. Some additional articles of interest were selected from reference lists of included articles

Only those articles were eligible to be included which were related to onset, development and progression of atherosclerosis and having roles in aging, vascular homeostasis, vascular cell differentiation, vascular development, gene expression, cell survival, growth and proliferation, and also contribute to atherogenesis. Screening of the literature was also done on the same basis. Data related to atherogenesis was extracted. When extracting data, mechanisms involving switching of vascular cells towards atherogenesis were primarily investigated.

Literature search began in May 2016 and ended in January 2019. During revision, further literature was searched and referenced by December 2019.

The literature search and all sections of the manuscript were checked multiple times during 11 months of revision February 2019 – December 2019 to maintain the highest accuracy possible.

The prime focus of the literature search was to screen the literature on the basis of eligibility criteria. Publications only in ‘English’ were used and there was no limitation on date of publication. Data extraction was based on these eligibility criteria. No unpublished study was used or included. This manuscript is a systematic review and adheres to relevant PRISMA guidelines. This research study finds evidence through already published research literature to investigate the atherogenesis and development of atherosclerosis in relation to pro-atherogenic pathological changes in the vasculature.

## Results

A total of 2894 articles were identified using database searching, 2682 were recorded after duplicates removal. Two thousand three hundred sixty-three (2363) were excluded after screening of title/abstract, 189 were finally excluded (because when many separate articles were present with similar conclusions, then only those were selected to be included which mainly focused on mechanisms contributing to atherogenesis), 5 articles were excluded during data extraction.

Finally 125 articles were included (because they had key implications towards switching of vascular cells towards atherogenesis).

*This study investigates the some hallmark changes in vasculature that result in ‘switching’ of vascular cells towards atherogenesis and contribute to development of atherosclerosis. The limitations of the study are defined in respective sections.*

### Pro-atherogenic changes in the biology of vascular cells that ‘switch’ the vasculature towards the development of atherosclerosis

In the process of atherosclerosis development, multiple factors play crucial roles. These factors include ageing, genetics, epigenetics, gender, vascular stressors such as turbulent blood flow, diabetes, hypertension, hyperlipidemia, smoking and others.

The role of smoking, hypertension, hyperlipidemia, diabetes and other risk factors is already well established and exploring them is beyond the scope of this study. But this study is concerned mainly with the changes that occur in vasculature and are involved in ‘switching’ the vascular cells towards atherogenesis.

**In this study, such pro-atherogenic changes in biology of vasculature are explored in relation to:** ageing, decline in vascular homeostasis, contributions to pro-atherogenic microenvironment caused by stem cell exhaustion and niche impairment. Chronic inflammation is also an integral part of atherosclerosis. This study also focuses on interconnectedness of atherogenic changes in vasculature that fuel the disease development. This study aims to focus on pro-atherogenic changes in vasculature largely in relation to ageing. In the latter part, it addresses the significance of dysregulated signaling pathways in atherogenesis. It also highlights the importance of interactions and cross talk between different factors involved in atherogenesis.

It also explores briefly the parallels between atherosclerosis and Alzheimer’s disease, as the risk of both the diseases increases exponentially with the increasing age. Atherosclerosis in neonates is also explored briefly as in depth investigation is beyond the scope of this study.

This study also points towards the significance of changes in gene expression and impairments in vascular homeostasis, contributing to the development of atherosclerosis. In the light of scientific literature, this study also postulates some hypothesis highlighting the switching of vasculature towards atherogenesis.

1. **Ageing and decline in vascular homeostasis contributing to the pro-atherogenic switching in vasculature**
  Ageing is involved in declining physiologic integrity, contributing to abnormal functioning and increased risk of disease development. It is already considered that there is interconnectedness between the hallmarks of ageing [5].
  The role of ageing towards the development of atherosclerosis is also very significant. Atherosclerosis is due to a vascular state that is increasingly pro-inflammatory, vasoconstrictive, proliferative and pro-coagulative. Ageing impacts vascular homeostasis profoundly and in turn contributes to switching of vasculature towards atherogenesis [6].
  Cardiovascular diseases are largely age related diseases. Vascular endothelial cells play a very significant role in vascular homeostasis. They act in autocrine as well as in paracrine fashion. Their dysfunction is involved in reduced endothelial dependent dilation. This contributes to decreased bioavailability of NO due to reduced availability of Tetrahydrobiopterin, all leading to lower vasodilation [7].
  The apoptotic pathways are upregulated in vascular endothelial cells with subsequent accumulation of ROS. There is also upregulation of: NADPH oxidase, ET – 1, NF-κB related pro-inflammatory signaling and transcription factors, vascular inflammation, advanced glycation end products (AGEs) promoting vascular fibrosis, CRP, IL – 6, ICAM, ACE, TNF alpha, MMPs, MCP – 1. There is dysregulation of several factors and pathways, they will be discussed later. Several factors including dilatory PGs, NO sensitivity, vitamin C, estrogen receptor alpha, vitamin D receptor and others which normally increase the endothelium dependent dilation, their functioning becomes abnormal [8] [9]. VEGF, Notch, wnt signaling and several other factors are also affected in the pathogenesis of atherosclerosis.
  *This paper will address them in the later part. Vascular Smooth Muscle Cells are addressed in later part in different sections. The role of ageing in atherogenesis is further explored under multiple headings.*
2. **Stem cell exhaustion and niche impairment, contributing to pro-atherogenic microenvironment and fueling the atherosclerosis development**
  The factors that are considered as key features of ageing such as stem cell exhaustion, niche impairment, chronic inflammation, epigenetic alterations, dysregulated cell signaling pathways and others, they are also involved in causing pro-atherogenic changes in vasculature [10]. These factors also contribute to the development of pro-atherogenic microenvironment. Ageing related DNA damage contributes to stem cell exhaustion. It also alters gene expression, transcriptional pathways and homeostasis [11]. With ageing, there is progressive decline in stem cell functioning including adult, vascular, mesenchymal and hematopoietic stem cells [10].
  There is also increased depletion and impairment of stem cell niche. This contributes to impaired microenvironment [12]. Stem cell niche plays role in regulating cell fate. Niche is involved in the tissue maintenance, generation, homeostasis regulation and repair. Niche also contributes to prevent stem cell depletion but the process of ageing contributes towards the depletion and impairment of both [13, 14]. All these factors are also pathologically altered in atherogenesis as well. This makes the role of niche impairment more significant.
  The stem cells discussed here are mainly mesenchymal stem cells. Stem cell ageing is considered to be involved in the development of atherosclerosis and other age related diseases such as Diabetes type 2, Alzheimer’s disease and others [15, 16]. Stem cells release paracrine factors and activate other stem cells. This is impaired by the pathologic changes in microenvironment. Microenvironment is involved in modulating cell biology and tissue response. The impact of paracrine factors such as NO, VEGF, FGF, HGF, angiopoietin, range from neovascularization to proliferation, migration, ECM synthesis and maturation [17]
  When adult stem cells are transplanted into the atherosclerotic vasculature, this increases the survival, proliferation, mobilization, differentiation, endogenous cardiac progenitors and also changes the vascular microenvironment. They also restore niches of stem cells that were previously in damaged or were in depleted states. Paracrine modulators interact with both the normal and pathologic cells of parenchyma and stroma. Stem cells also release factors that function in autocrine manner and induce the cell growth, renewal, survival and regulate cell’s own fate, stemness, behavior, commitment lineage and their own biology. Ageing and disease development mechanisms alter the gene expression which results towards changes in functional properties of cells. In case of stem cells, this change impacts self-renewal and lineage potential [18] [19].
  In hypoxic states, vascular stem cells upregulate the release of cytokines, chemokines and growth factors several folds. They also induce vasculogenesis and cytoprotection. In hypoxic states, vascular stem cells also significantly upregulate the expression of VEGF, FGF, IGF 1, HGF, adrenomedulin, thymosin beta 4, SDF 1, PDGF, IL 1 beta and others [20]. AKT 1 expression is also upregulated, it interacts profoundly with PI3K, mTOR, shh, EGF, FOXO, CREB, PTEN, and apoptotic pathways. The pro-survival genes are among the key upregulated genes, they provide cyto-protection via interacting with wnt signaling pathways.
  Multiple cellular pathways, processes, molecules and mediators interact with each other in the process of disease development. Signaling factors and pathways that confer cytoprotective effects, they act through PI3K/AKT pathway, protein C kinase, ERK ½, STAT 3, and it is assumed that several other pathways and mediators are also involved which are still unidentified. FGF 2, EPO, SFRP 2 and Tetrahydrobiopterin also confer cytoprotective effects [21, 22].
  *It may be hypothesized here that the role of microenvironment is very crucial in onset, development and progression of atherosclerosis as the changes in gene expression keep unfolding; the microenvironment continues to move towards more atherogenic state. The role of stem cells in this aspect is very crucial as they express proangiogenic, proarteriogenic paracrine mediators in temporo-spatial manner. Their effects are different in different conditions depending on microenvironment after injury.*
  *Stem cell niche contributes to the maintenance of adult stem cells and when they are disrupted, this disruption damages and kills adult stem cells. The impairment of vascular stem cell niche contributes to the impairment of vascular microenvironment and vascular stem cells. Vascular stress that imparts its effect on cell biology, genetics and epigenetics of vasculature with increasing age, is one of the key components that play role in atherosclerosis development. Physiologically, adult heart vessels are quiescent. It is in pathology or stress conditions, there is expansion of vascular bed.*
  *It may also be hypothesized here that the functioning of paracrine factors has limitations which are dependent on the maintenance of therapeutic concentration in time dependent manner. Their effect on cells also depends on both these factors; niche maintenance and ageing. In disease microenvironment, paracrine factors also are unable to induce their physiologic positive effect because there are so many factors that are playing their roles such as inflammatory mediators and interactions among them play crucial roles in disease progression. The pro-atherogenic mechanisms involved in disease development, they interact with each other in terms of gene expression and work in interconnected manner in ways that impact gene expression, epigenetics and vascular cell biology.*
3. **Role of Chronic Inflammation in switching vascular cells towards atherogenesis**
  The role of inflammation is very crucial in atherosclerosis. It is involved in endothelial dysfunction, accumulation of LDL and macrophages, foam cell formation, fatty streak accumulation, smooth muscle cell migration, all leading to complex atheroma formation. It also involves the release of cytokines, growth factors and inflammatory mediators.
  Chronic inflammation is involved in the development of many diseases including atherosclerosis. It plays a central role via release of TNF alpha, IFNs, stromal cell derived factor 1, monocyte chemoattractant protein 1. The process of atherogenenesis is further potentiated by release of altered growth factors and cytokine milieu, ROS and hypoxia [23].
  Development of hypoxic niche due to VSMC proliferation and expansion further accelerates the progression of atherosclerosis. All these factors work together to activate gene networks that enhance survival in such vascular environment. Obesity is involved in many diseases including metabolic syndrome and is also involved in increasing vascular inflammation. The risk of metabolic syndrome increases with ageing. Systemic vascular inflammation increases with ageing and release of several pro-inflammatory mediators such as IL -1, IL - 6, IL-1β, IFNs and several other mediators [24].
  The process of atherosclerosis is a complex process in which pattern recognition receptors present within cells are activated. In this aspect different regulatory events play crucial role involving RNA, DNA, ATP, ECM modifications, mitochondira and HSPs.
  It is well established that mechanisms that control inflammation, become dysregulated with ageing. Senescent cells accumulate with age and contribute to secreting inflammatory molecules which in vascular cells contribute to atherogenesis. The senescent associated changes in phenotype are also responsible for shifting the gene expression towards pro-atherogenic genes. This ultimately potentiates the development of chronic low grade inflammation. Endogenous pro-inflammatory signals accumulate with ageing. Ageing is associated with increase in systemic levels of inflammation. This involves upregulation of inflammatory mediators including TNF alpha, caspase 1, IFN gamma, IL-1 beta, IL-6, IL-17, CRP, C5a, C3a receptors, inflammatory chemokines and adipokines. These mediators are also involved in obesity related upregulation of inflammation. NF-κB upregulation has profound impact on this process [25–27].
  NF-κB which is associated with controlling many genes that are involved in inflammation, it becomes highly upregulated in atherogenesis. This upregulates inflammatory pathways several folds and potentiates the process of atherogenesis [28].
  The continuous evolving nature of atherosclerotic plaque keeps changing the gene expression of vascular cells towards pro-atherogenic. Chronic vascular inflammation progresses as cascades and causes the activation of endothelial cells. It also upregulates the gene expression of: intracellular adhesion molecules, VCAMs, integrins, selectins and others.
  VSMCs proliferation potentiates narrowing of vessels and role of hypoxic niche is very crucial. This involves sequential shift towards the gene expression of hypoxia inducible factors, glycolytic enzymes and VEGF expression. The Nod like Receptor (NLR) family is also important in ageing. It increases its expression in age related systemic inflammation and also increases Caspase – 1. It upregulates levels of IL − 1 beta and IL − 18. Inflammasome NLRP3 is upregulated in response to accumulation of amyloids, excess glucose, urate and cholesterol crystals. It also contributes to the insulin resistance, and the role of insulin resistance in promoting atherosclerosis is already well established. NLRP3 contributes significantly in atherogenesi s[29].
  *It is hypothesized here that chronic inflammation is directly involved in pro-atherogenic gene expression change, dysregulations in vascular micro-environment, alterations in genetic and epigenetic homeostasis, dysregulations in cell circuitry and alterations in vascular homeostatsis. This study focuses on biological changes that are involved in switching of vascular cells towards atherogenesis. From vascular lipid deposition, fatty streak formation to formation of atheroma, all stages involve sequential changes in gene expression of vascular cells. This process involves complex pathological cross talk between different cell types which alters cell signaling pathways such as VEGF, wnt and others. This is addressed in detail in the later part of this study.*
  *All the inflammatory cascades playing role in atherogeneisis, they further disrupt the vascular homeostasis and further fuel the development of atherosclerosis.*
4. **Dysregulated signaling pathways alter vascular homeostasis and contribute to the disease progression**
  In this section, we investigate how dysregulated signaling pathways such as Wnt, Hedge hog and Notch signaling pathways contribute to the switching of vascular cells towards atherogenesis. It is predicted here that all the signaling pathways and cell circuitry work in a network of complex interactions that’s dependent on concentration, microenvironment, cell type and sequential time dependent manner. In disease development, all these are impaired, their interactions and cross- talk further fuel the disease progression.

#### Wnt signaling

Wnt family involves 19 genes that are highly conserved and essential for embryogenesis and development. They play key regulatory roles in cell differentiation, proliferation, migration, polarity, cellular behavior (through transcription factors, NF Kappa beta and others), cell cycle activation, survival (IGF − 1), regulation of ECM and MMP, calcium release from intracellular stores and in activating co-effector kinases [30]. The Wnt regulated cellular behaviors include cytoskeletal organization, cell polarity and cell motility. Cross talk between pathways is also based on the cell types and corresponding receptor expression. Pro-proliferative genes such as cyclins are regulated by Wnt beta catenin signaling in VSMCs proliferation. In VSMCs proliferation, there also is down regulation of P21. Dysregulations in Wnt signaling lead to neointimal thickening [31, 32].

The proliferative effect of Wnt is lost with increasing age despite sustained Wnt signaling. Wnt signaling also plays role in cell fate specification and lineage commitment. When signaling pathways become aberrant, the alterations contribute to switching vasculature towards atherogenesis. The cell biology of vascular cells begins to deviate from cell type specific gene expression, this further progresses the atherogenesis. The role of pathologic interactions is also very significant as Wnt proteins interact with calcium, JNK, GSK 3beta, CREB transcriptional factors. They play role in atherosclerosis and other age related diseases such as Alzheimer’s and different cancers [33]. In embryogenesis, Wnt signaling is an important regulator of: VSMCs differentiation and morphogenetic signaling gradient. It also controls regeneration. There is diminished expression of Wnt pathway co-receptor LRP 5/6 in atherosclerotic plaques. Any mutation in LRP 6 gene increases risk of coronary artery disease, hypertension, diabetes mellitus type 2, also increases levels of LDL, cholesterol, DKK 1, and also there is abnormal upregulation of inflammation [34, 35].

The role of Wnt signaling in disease mechanisms is not limited to atherosclerosis but extends to DM, breast cancer development, Alzheimer’s and several other diseases. Wnt signaling is very crucial as it plays numerous roles in multiple key processes including cytoskeletal regulation, cell fate specification and calcium based signaling. It interacts with NF-κB, Shh, JAK – STAT, TGF beta, FGF, notch signaling, retinoic acid, cytokines such as IL – 6, LDL, several other regulatory genes and transcription factors. Wnt signaling also amplifies mechanical signaling that is involved in embryogenesis [36].

It is important to remember that oscillatory blood flow is one of the main factors that play key role in switching the vascular gene expression in vessels towards atherogenic. The pathologically altered signaling pathways interact and cross talk with different contributors involved in atherogenesis such as inflammation. This shifts the gene expression to become more pro-atherogenic [37, 38].

In Atherosclerosis, there is upregulation of: VSMCs proliferation, endothelial apoptosis and cellular migrations. Wnt proteins are regulators of VSMCs behavior through beta catenin dependent and independent Wnt signaling pathways. Endothelial injury and dysfunction leads to de-differentiation of VSMCs [39].

*It is hypothesized here that this switch towards de-differentiation is also a phenotypic manifestation of deviation from differentiated state of vascular cells. It also points that atherosclerosis originates when extracellular agents, inflammatory mediators, ROS and other oxidative stressors, oxidized LDL, cytokines such as IL 1 beta, IFN gamma, all these and other mentioned above factors alter the vascular microenvironment. This also changes gene expression and alter cell biology of vascular cells. When such damage accumulates beyond a specific level, this leads to the phenotypic onset of the disease. Aging is one of the most significant factors in the entire process of disease development.*

*Age related alteration and changes in Wnt signaling may trigger a cascade of events that undergo to switch the gene expression of vascular cells towards disease development and further disease progression.*

*In this aspect, the role Hh and Notch signaling pathways is also very crucial.*

#### Hedge hog signaling

Shh plays diverse and crucial roles both in embryonic development and post-natal homeostasis [40]. Its role extends from induction of angiogenesis to repair and to regeneration of ischemic myocardium and skeletal muscles. Diabetes negatively impacts Shh signaling. Hh pathway is highly conserved, essential for cell fate decisions, homeostasis of adult tissues [41, 42]. The mechanisms and role of Hh is not entirely known in neovascularization but evidence points it to be very crucial. Mononuclear blood cells play role in angiogenesis and arteriogenesis in ischemic tissues through cytokines, growth factors and proteases, as Shh is a potent chemoattractant for human monocytes among other cells. Hh provides guidance, promotes neural migration and migration of endothelial progenitor cells. Hh pathways also interact with several other pathways and proteins such as PI3K pathway, G proteins and others [43, 44]. VE cells, VSMCs all express Shh, it is also expressed increasingly at sites of inflammation and repair. Hh expression is involved in tumorigenesis and ischemic injury. Obesity, DM, systemic inflammation all are involved in dysregulation of Hh signaling and speeding up pathogenesis of atherosclerosis [45, 46].

Healthy vessels have higher levels of Hh signaling while atherosclerotic vessels have dysregulated and decreased levels of Hh signaling though Hh ligand is present in plaques. Hh signaling is critical for VEGF production, endothelial maintenance and growth. It is important to remember that LDL acts like antagonist of Hh pathway [47].

When Hh signaling is downregulated, it contributes to progression of ageing and age related diseases such as atherosclerosis, type 2 diabetes, osteoporosis and Alzheimer’s disease [48, 49].

*It is postulated here that Hh signaling plays a cascade of roles that are very essential for vascular homeostasis. Alterations in Hh expression due to pro-atherogenic changes in vasculature, further fuels the development of atherosclerosis.*

#### Notch signaling pathways

Notch genes are evolutionarily conserved and are involved in intracellular signaling mechanisms, angiogenesis, vascular modeling, vascular homeostasis and others. Different Notch genes interact with each other to play their roles. Mutations in this pathway disrupt cell fate determination and homeostasis [50]. Notch genes interact with several other genes and pathways including Jag 1,3, Dll 1, 3 genes, Ephrin/Eph pathways and others. In the process of vasculogenesis and angiogenesis, there are several pathways that play key roles such as VEGF, TGF beta, the angiopoietin/tie receptor pathway, ephrin and its receptor pathway. Notch is significantly expressed in VE cells, is also key regulator of vascular morphogenesis and angiogenic vascular modeling [51, 52].

Notch signaling plays important roles in modulation of cell proliferation, survival and stem cell maintenance. Dysregulation of this pathway contributes to atherosclerosis progression and development as Notch signaling is involved in CVS development and its maintenance in adult life. It also is fundamental for cell fate determination. It interacts with the regulators of cell cycle, apoptosis, inflammatory cytokines, NF-κB, estrogen, VEGF receptor, also interacts with negative regulators of transcription such as Hes and Hey gene families [53].

Oscillatory blood flow which is one of the key steps in vascular switching towards atherogenesis, also impairs vascular Notch signaling and interferes with the expression of pro- survival genes, anti-inflammatory genes, endothelial cell dysfunctions, pro-apoptotic genes. Notch signaling also counters IL 1 beta based VSMCs trans-differentiation. It interacts with DII4, Jagged 1 and inflammatory mediators. Ligands of signaling pathways are encoded by different genes such as in Notch by delta and serrate genes. At the cellular level, there are multiple genes that all work together to produce a specific phenotypic effect properly [54–56].

*Hence, atherogenesis is a complex multifactorial process that unfolds like an interconnected loop of disease progression. It is hyothesized here that when some of the genes involved do not work properly then this leads towards abnormalities in that pathway and when such changes in gene expression have accumulated significantly, then the cell switches to express its abnormalities in the form of disease development.*

*It is postulated here that in the microenvironment of disease, alterations in cell signaling cause worsening of atherogenesis. This happens in a series of unfolding pathological events that cause changes in vasculature. Such changes include activation of Notch in atherosclerotic plaques and accumulated macrophages, resulting in worsening of the inflammation and atherosclerotic plaque progression.*

*Notch protects endothelium from dysfunctions caused by inflammatory cytokines. They (cytokines) through NF-κB inhibit Notch signaling, induce apoptosis, upregulates CAM, downregulate endothelium dependent NO synthesis.*

*It is important to note that VSMCs are the cells that are not terminally differentiated, they under inflammatory conditions switch from a contractile quiescent to secretory and proliferative state which leads to secretion of inflammatory molecules. This phenotypic switch induced by predisposing factors such as ageing, oscillatory blood flow and others that disrupt the epigenetic homeostasis are at the core of atherosclerosis disease development. These changes including dysregulations in vascular homeostasis, signaling pathways and others, together they result in switching of the vascular cells towards atherogenesis.*

5. **Interactions and cross-talk among disease development mechanisms involved in atherogenesis**
  There is already emerging evidence about the interconnectedness of genes in the form of gene networks that are disease specific [57]. Atherosclerosis is a chronic inflammatory complex disease. It is associated with severe changes in gene expression in the vascular and other related cells that play role in pathogenesis of atherosclerosis. The alterations in gene expression include: osteoclast associated receptor that is involved in call activation and inflammation, cytokine interactions such as ILs, TGFs, IFN, TNFs, NF-κB. These upregulated genes are mainly involved in immune system processes. The genes that are downregulated are the ones that take part in cytoskeletal organization, organization of cellular components and regulation of cellular processes. Such changes in epigenetics alter vascular homeostasis. Genes involved in atherogenesis impact, interact and cross talk with each other directly or indirectly [39]. VEGF is downregulated with aging. This contributes to impairment in angiogenesis. This downregulation also causes an imbalance between pro-angiogenic VEGF and anti-angiogenic endostatin. VEGF also work through interactions with several crucial gene products such as P-STAT 2, P – CREB. Both these transcription factors work in association with HIF 1 alpha to upregulate VEGF gene expression [58].
  There are several genes that are atheroprotective; their functioning is largely disrupted by vascular stress in vascular endothelial cells. Such genes are also influenced by and interact with other gene products such as cAMP [59, 60]. Pro-atherogenic changes in vasculature further fuel the disease progression such as upregulation of apoptotic pathways make the vessel wall highly pro-coagulative [61].
  The signal transduction mechanisms are directly impacted by oscillatory vascular stress which shifts vascular epigenetics towards atherogenesis. It also upregulates MAPK which is responsible for transmembrane signal transmission for cell growth and differentiation. This change in gene expression of VSMCs also leads to the upregulation of PDGF receptor. This oscillatory vascular stress also impacts cell surface and alters receptor conformation [62, 63].
  In atherosclerosis, vascular endothelial cells and smooth muscle cells both are pathologically altered in a complex manner such as there is increased apoptosis in vascular endothelial cells while hyperplasia in VSMCs [64].
  *It is hypothesized here that there are different phases of atherosclerosis development and the role of different gene expression products also varies with different phases of disease development. Its impact is based on vascular microenvironment. Inflammation, oscillatory blood flow and ischemia, all induce apoptosis in vascular endothelial cells. TNF alpha, IL 1 Beta, NF-κB and other pro-inflammatory factors dysregulate signaling pathways such as NOTCH. These factors are pro-atherogenic. The role of key signaling pathways that are also involved in embryonic vasculogenesis and are also dysregulated in atherosclerosis is very crucial in many aspects. It is postulated here that this interconnected loop of pro-atherogenic vascular changes alters the gene expression of vascular cells. It also makes the microenvironment, cell signaling pathways, cell fate and vascular homeostasis maintenance mechanisms dysregulated.*
  *In the process of disease development, the pathologic interactions between different genes and their gene expression products result in disrupting the cell fate related regulatory genes and vascular homeostasis. With increasing age, this changes the cell biology/homeostasis to such an extent that the disease begins to express itself. What causes the onset of all these pathologic changes that lead to the disease development? One of the most significant factors is ageing. This is discussed and further explored in later part of this study.*
  *All the hallmarks of ageing working together, they accumulate the damage in vasculature and disrupt the vascular homeostasis to such an extent that this contributes to the switching of vascular cells towards atherogenesis*
6. **Role of dysregulations in homeostasis of vascular cell types in the development of atherosclerosis**
  Each cell type is expression of particular subset of genes that is responsible for particular pattern of gene expression. This particular expression is established through gene regulatory networks. Interactions between cell type specific subset of genes are also responsible for morphogenesis and cellular differentiation [65].
  Cellular differentiation is based on modifications in gene expression and transforms a cell into the specifics of cell type. This process changes cell size, cell shape, membrane potential, membrane activity and signal responsiveness [66]. It is considered that there is some combinatorial code that is responsible in establishment of cell types. When a cell deviates from its differentiated state, this is more likely to be due to changes in epigenetics, cell biology and gene expression that deviate a cell from its differentiated state and ultimately from cell type specific combinatorial code [67]. In the process of atherogenesis, de-differentiation of VSMCs and endothelial dysfunction are very crucial in this aspect [39].
  In the process of cellular differentiation including during development too, cell signaling pathways alter epigenome. The process changes gene expression profiles by turning off stem cell genes and by activation of cell fate genes [68]. Wnt is involved in all stages of differentiation. Depletion of growth factors such as BMPs, TGFs, and FGFs promotes embryonic stem cell differentiation. Shh promotes differentiation, Notch is involved in proliferation and self-renewal of stem cells. Retinoic acid promotes differentiation [69, 70]. Signaling pathways also induce epigenetics based alterations in cell fate; their precise role in this aspect is largely unknown. The role of microenvironment is unique to different cell types. Changes in microenvironment via alterations in mechanotransducer mechanisms contribute to alterations in epigenome [71]. Differential regulation and maintenance of specific regulatory genes is required for cell fate selection and maintenance. Concentration of such gene products guides the cells towards specific paths and precise balance of their concentrations is also required for cell type homeostasis and maintenance. Alterations in their gene expression impact cascades of other inter-related genes and differentiation of cells. Epigenetic mechanisms are also the key regulator of cell fate through regulating patterns of transcription [72].
  All the altered factors such as signaling pathways, gene expression, epigenetics, dysregulations in cell biology, genomic instability disrupt vascular homeostasis, contribute towards deviation of vascular cells from their differentiated fate. The greater this deviation is, the more adverse the atherogenesis becomes [73]. Hypertension, DM, smoking, alcoholism, sedentary lifestyle, obesity, high cholesterol, high triglycerides and all those factors that cause vascular stress are involved in dysregulating the combinatorial code [74, 75].
  *It is postulated here that the cell type specific gene regulatory networks become altered in atherogenesis. Such genes include Hox genes that are also developmental master regulators and work in tissue specific spatio-temporal manner. With ageing, there is significant decline in the impact of Wnt signaling pathways despite sustained signaling. Other pro-atherogenic changes in vasculature are postulated to be due to dysregulations in cell circuitry, alterations in epigenetic tags and accumulation of pro-atherogenic changes in gene expression.*
7. **Impairment in maintenance of vasculature: Role of Hox and some other proteins**
  Hox genes are involved in maintaining vessel wall integrity, homeostasis, vascular remodeling upon disruption. These genes interact reciprocally with NF-κB. Hox genes have persistent post-natal expression, they are also involved in angiogenesis. Hox expression varies in different parts of vascular bed, that’s why; some parts are more or less prone to CVD development. These genes also have wide range of interactions ranging from FGFs to integrins. Hox genes work in regulatory networks. They are genes of diverse functioning such as ECM modeling, transmembrane signaling, cell cycle control, transcription factors and inflammation in VSMCs and ECs. It is considered that there is a vascular Hox genes based Code which when dysregulated, contributes to atherosclerosis development [76, 77]. Hox genes are involved in morphogenetic events, differentiation, regulatory self-renewal and differentiation of stem cells. They regulate differentiation of vascular wall stem cells into VSMCs via epigenetic mechanisms. Dysregulations in vascular wall stem cells also play role in atherosclerosis and neointimal plaque formation. The functioning of Hox genes is very crucial as they are master regulators of regional specification and organ development. Hox proteins are transcription factors that work with co- factors to activate or repress target genes. They regulate lineage specific gene expression. They also interact with several kinases such as MAPK, extracellular regulatory kinases and others [77, 78]. Hox genes are highly conserved, important in vascular development; they are controlled by DNA methylation and are dysregulated in cancer [79, 80]. In this aspect, Hox genes are also very important as they are involved in vascular remodeling, disease related changes in gene expression, also impact ECM and integrins. HoxA5 is endothelial function regulator and is involved in migration, inflammation and angiogenesis. CAMP response elements (CRE) in relation with HoxA5, Klf3, Cmklr 1, Acvrk1, spry2 and other genes, serves as mechanosensitive master switch in gene expression [81].
  Cells in vascular bed are quiescent and in vascular beds there resides niche for stem cells. Pathogenesis of atherosclerosis also disrupts the vascular stem cell niches and damage stem cells. This further fuels the disease progression [82–84].
  *This signifies the role of switching in gene expression towards the disease development. It is hypothesized here that atherosclerosis originates due to dysfunction in homeostasis of vascular cell types such as ECs, VSMCs, immune cells and fibroblasts. This onset is consequence of alterations that first begin with sensitive genes and accumulate over time, leading to cascade of alterations extending over wide range of genome.*
8. **Epigenetic alterations in the development of atherosclerosis and the role of oscillatory blood flow**
  Epigenome maintains cell type specific gene expression throughout differentiation. It also plays role in ECs homeostasis and vascular development. Epigenetics change greatly during the process of differentiation. Epigenetic changes become stable, epigenetic tags of terminally differentiated cells begin to govern gene expression and function. Oscillatory blood flow is involved in switching gene expression towards atherogenesis, impacting greatly vascular ECs. Stable blood flow reinforces protective gene expression. There are several mechano-sensitive genes such as HoxA5, Klf3, Klf4 that are also involved in vascular development, remodeling and in disease development. Stable blood flow upregulates Klf 4 gene which acts as anti-inflammatory and maintains gene expression in quiescent ECs. Oscillatory blood flow upregulates DNMT3A gene, its function is opposite of Klf 4 [85, 86].
  Genome involved in any specific cell type code, works as interconnected and inter-related network of genes. Epigenetic alterations in cell type specific combinatorial code pathologically influence the functioning of vascular gene expression. The regulatory networks of genes in vascular cells are involved in a network of functions; ranging from regulation of endothelial cell biology to homeostasis. When they are epigenetically altered, it contributes to disease development. Atherosclerotic changes are so profound that they even impact internal architecture of the affected cells such as loss of desmin, appearance of vimentin expression. Oscillatory blood flow upregulates pro-atherogenic genes, results in chromatin remodeling, alters gene expression of ECs which through mechano-receptors initiate a cascade of signaling events that leads to sequential development of atherosclerosis. Dysfunctional ECs phenotype initiates and propagates atheromatous plaque accumulation [87, 88].
  Atherosclerosis shares many phenotypic characteristics with cancers such as hyperproliferation, migration, inflammation, alterations in epigenetics [89, 90]. Blood flow mediates the functioning of TFs. It also alters or maintains the epigenetic tags in vascular cells depending on pace of blood flow. All these factors interact and cross-talk with each other to regulate vascular cell biology. Oscillatory blood flow, smoking, systemic inflammation, sedentary life style all contribute to atherogenesis as risk factors [91]. It is the changes in vasculature that switch the vascular cells towards atherogenesis.
  *It is hypothesized here that there is an internal genetic and epigenetic homeostasis that is established at the time of establishment of cell fate. When this homeostasis is dysregulated due to pathological factors altering gene expression, it then sets the affected vasculature towards the route of atherogenesis.*
  *Such changes set in motion a cascade of disease unfolding mechanisms that result in endothelial dysfunction, de-differentiation of VSMCs, alterations in intermediate filament content, chronic inflammation and inflammatory mediators. It also points that there are cascades of atherogenic changes in cell circuitry and gene expression, ranging from alteration in signaling pathways to alteration in transcription factors, such changes play key roles in development of atherosclerosis. The competence; ability to respond to signals, of cells is altered with increasing age. This ability is different in every cell type. Atherogenesis also impairs the competence of vascular cells, resulting in decline in vascular homeostasis.*

Here we briefly explore the Atherosclerosis in relation to Alzheimer’s disease and Neonatal Atherosclerosis, as in depth focus here is beyond the scope of this study.

### Some key parallels between Alzheimer's disease and Atherosclerosis

The risk of both Alzheimer’s disease and atherosclerosis increases exponentially with increasing age [92]. One of the most intriguing features between the two diseases, are the many parallels in terms of disease development mechanisms. We briefly explore them here as the in depth investigation of such mechanisms is beyond the scope of this study.

In atherosclerosis, atheromatous plaques accumulate in blood vessels. In AD, there is accumulation of amyloid plaques and NFTs. AD is also associated dysfunction of vascular cells. In AD, there is profound deposition of amyloid plaques resulting in vascular endothelial damage. Endothelial dysfunction and death plays key role in atherosclerosis, and there is increased neuronal death in Alzheimer’s disease [93, 94].

Both Atherosclerosis and Alzheimer’s disease involve thickening of vascular walls and occlusion. Chronic inflammation plays key role in both the diseases involving profound role of inflammatory mediators, signaling molecules and inflammation related gene expression changes [93, 95, 96].

In both the diseases, there is upregulation of p53, p21, Bax, apoptotic pathways. There is down regulation of IGF – 1, Bcl − 2, NGF, BDNF, neurotrophins, growth factors and signaling proteins [97, 98].

Diabetes Mellitus type 2 is also a common factor that contributes to the development of both atherosclerosis and Alzheimer’s disease. EGFR is involved in upregulation of VEGF signaling through PI3K and MAPK pathways. It also becomes dysregulated in both the diseases. This dysregulation of EGF along with its receptor is also involved in neointimal hyperplasia [99–101].

Both diseases are associated with pathological alterations in the microenvironment. Microenvironment alterations contribute to infiltrating monocytes and macrophages, upregulation of ROS through upregulation of NOX 1 is also involved in promoting progression of atherosclerosis. It is important to note that ROS are also involved in the pathogenesis of Alzheimer’s disease. ROS downregulates NO based vasodilation and promotes pro-inflammatory signaling [92, 102]. There is also upregulation of stress activated protein kinases in relation to p53, APP, dysregulation of ubiquitin protease mechanisms, intimal deposition of amyloid. TGF beta plays crucial roles in cell differentiation. Its role is complex in both AD and atherosclerosis. Its inhibition accelerates atherosclerosis. It plays cytoprotective role via modulating inflammatory response and lipid accumulation. HDL also induces expression of TGF beta [103–105].

Estrogen in many studies has been shown to be protective against atherosclerosis as well as AD [106, 107].

Systemic inflammation is also common contributory factor in the development of atherosclerosis and Alzheimer’s disease [108]. APOE is also involved in both the diseases. APOE variant E4 is highly involved with increased risk of disease development for both AD and atherosclerosis [109, 110]. Aβ and atheroma, both are toxic to vascular endothelium. There is a very strong immune component associated with AD and atherosclerosis involving similar inflammatory mediators such and CRP, TNA alpha, IL-1 and others [111, 112].

*It is postulated here that the development of Alzheimer’s disease is a multistep process just like atherosclerosis. The toxic microenvironment resulting in gene expression changes, abnormalities in cell signaling mechanisms and chronic inflammation are among the most significant parallels between the two diseases. The cellular homeostasis is disrupted in both the diseases. These are just brief outlines of the parallels between the two diseases as both diseases are also regarded as age related diseases.*

### Atherosclerosis in Neonates

The biological processes involved in atherosclerosis begin early in life and mostly it is with increasing age that atherosclerosis reaches to the level of clinical presentation and its pathological effects are pronounced [113]. Multiple factors play role in development of atherosclerosis ranging from genetic, behavioral to environmental factors. Pro-atherogenic conditions may be result of childhood obesity, familial syndromes, hereditary dyslipidemias, chronic inflammatory diseases or increasing age [114].

Atherosclerotic lesions have also been found in neonates. There are multiple factors that have been associated with neonatal atherosclerosis including maternal smoking, hypercholesterolemia, inflammatory diseases and others. Apolipoprotein E deficiency is considered to cause endothelial dysfunction in neonates [115–117].

It is also considered that in neonatal atherosclerosis the atherogenesis actually begins during prenatal development. This is considered to be due to changes in prenatal development environment. Such changes may be responsible to impact the epigenetics in development [118]. Several studies have also associated low birth weight in neonates with increased risk of atherosclerosis. Children born to diabetic mother are at increased risk for obesity later in life which is also risk factor for atherosclerosis development [119].

It is important to note that congenital heart defects related irreversible pulmonary hypertension is not associated with increased atherosclerosis risk, despite causing systemic endothelial dysfunction [120]. Cyanotic congenital heart disease patients have been found to have decreased risk of atherosclerosis [121–123].

In neonatal atherosclerosis, atherosclerotic changes range from myointimal mild thickening to soft early plaques and VSMCs hyperplasia. It has also been found that exposure to factors such as maternal smoking and hypercholesterolemia, interfere with the prenatal development and alter microenvironment. They induce pro-atherogenic changes in gene expression of aorta and other vessels [124].

The presence of atherosclerosis in neonates challenges the role of ageing and atherosclerotic development. It signifies that development of atherosclerosis is multifactorial and the role of ageing in atherogenesis is also very significant [1, 125].

*It is hypothesized here that dysregulations in cell signaling pathways such as NOTCH, Hh pathway, wnt and others that are involved in vasculogenesis and angiogenesis may have profound role in development of neonatal atherogenesis. They have also been found to play significant role in the development of atherosclerosis in adults. The earlier part of paper highlights their significance. The role of maternal smoking and maternal diseases in causing neonatal atherosclerosis should also be investigated at epigenetic level. Neonatal atherosclerosis may be a combination of pathologic microenvironment, epigenetic and genetic alterations ultimately impairing the maintenance of vascular homeostasis.*

*It is also postulated here that the lack of increased atherosclerosis risk in neonatal cases with pulmonary hypertension and decreased risk of atherosclerosis in congenital heart disease signify the role of chronic accumulation of pathological changes in vasculature. Such chronically accumulated damage impairs the vascular homeostasis by inducing pro-atherogenic epigenetic changes.*

*Neonatal atherosclerosis development is briefly explored here, as deeper investigation into the topic is beyond the scope of this study.*

## Discussion

The pro-atherogenic changes are the result of pathologic alterations in vasculature that dysregulate the vascular homeostasis. This impacts the vascular cells at epigenetic, genetic and cellular level, ultimately resulting in switching of vascular cells towards atherogenesis. In this aspect, the role of ageing is very crucial. It also causes dysregulation in regulatory genes and vascular homeostasis through alterations in epigenetics and genetics, signaling pathways, cell circuitry, genome instability, heterotypic interactions between multiple cell types and vascular microenvironment. Such pathological changes in vasculature play key role in the process of atherogenesis. This study focuses on the ‘switches’ that are involved in causing the pathological changes in vasculature and hence result in atherogenesis.

The particular sequence of unfolding of any disease is also determined by the sequential step by step interactions among dysregulated regulatory genes, signaling pathways, gene expression products, growth factors and others. This particular sequence is cell type specific as the epigenetics of every cell type varies.

It is already considered that there is a combinatorial code (set of regulatory genes) that is involved in development and determination of cell types. With ageing, genomic instability also increases. It occurs due to alterations genome, its maintenance, and accumulation of epigenetic alterations, malfunction of p53 and other regulatory genes. Different sites of genome become more susceptible to aberrations such as amplifications and deletions. Such alterations cause widespread destabilizations and fuel disease progression. In our genome, there are switches which are governed by opposing factors such as TSP - 1 and inducing factors such as VEGF – A and FGF among others. They also regulate: homeostatic survival of vascular endothelial cells, gene expression of Ras, Myc and other such key genes which upregulate angiogenic factors. The role of critical regulatory genes is very significant as their dysregulation, repression or over-expression impact profoundly towards shift in such switches.

Another most important pro-atherogenic change in vasculature is pathologic shift in microenvironment. In this aspect, inflammation plays very crucial role by providing bioactive molecules to disease microenvironment. The role of chronic inflammation in altering vascular microenvironment also signifies the role of alterations in vascular homeostasis towards atherogenesis.

The pathologic alterations in vascular homeostasis also impact the interactions and cross-talk among different genes, resulting in further alterations in the epigenetics of vascular cells. In order to understand the complexity of atherosclerosis onset and progression, we need to map the sequential changes in gene expression that ultimately lead to the development of atherosclerosis. This way disease specific map of changes in cell circuitry can be developed.

One very important finding in this study is the role of developmental pathways and mechanisms in disease development that is also very crucial in adult homeostasis. Their dysregulations have significant pathological impact in disease development; such as dysregulations in shh, wnt, Notch pathway and others in atherogenesis. The de-differentiation in VSMCs and endothelial dysfunction is also a consequence of this vascular switching.

It is postulated in this study that when the pathological alterations in vasculature accumulate beyond a specific threshold to the extent that they interfere with gene expression, regulatory genes, epigenetics, genetics and cell biology, then the vasculature switches to atherogenesis. The process of atherosclerosis is manifestation of such underlying changes. In the vascular cells affected by atherosclerosis, several disease antagonizing and promoting mechanisms come into play. This involves complex diverse alterations in cell machinery including dysregulations in interconnected signaling pathways; Notch, VEGFs, FGFs, angiopoietin. These pathways were involved in development of vascular cell types and in their maintenance postnatally. The disease affected cells when interact with other cells, they feed signals that contribute towards disease progression. The succession of changes in genome and worsening of microenvironment leads to emergence of disease. Depletion of vascular stem cell niches also contribute to pro-atherogenic changes in microenvironment.

This study may have profound implications and some are pointed here. This study may also potentially provide new insights into the biology of aging. This study may also contribute towards the development of new therapeutics by pointing to new strategies which involve restoring the epigenetics, gene expression and cell biology in vasculature towards the vascular homeostasis. Preventing the pathological switching of vascular cells by controlling the ‘switches’ in cell circuitry may lead to the development of new novel therapeutic approaches towards the prevention and treatment of atherosclerosis. This may also involve blocking the sequential unfolding of pathological mechanisms, signaling pathways, their interactions and cross-talk to halt the progression of vasculature towards atherosclerosis. For this purpose, utilization of gene editing techniques such as CRISPR-Cas9 should also be evaluated. This research article may also help in further answering the questions regarding onset, development and progression of Atherosclerosis.

This study may help in devising similar studies on other diseases. In the future, all the changes in vasculature that contribute to atherogenesis including decline in homeostasis and others, they should also be investigated in other diseases. There may have many parallels, similarities and common basis in disease development among multiple diseases which have not yet been identified. This approach should first be adopted for age-related diseases such as Alzheimer’s disease, atherosclerosis, osteoporosis, cancers, Idiopathic pulmonary fibrosis, neurodegenerative and others. The switching of gene expression and switches involved also need to be focused profoundly in such future studies.

In the light of scientific literature, this study also suggests some hypothesis while citing evidence in support of this study from already well-established and published research literature. Future research and experimental studies at molecular, genetic, epigenetic and cellular levels will reveal further details about the findings presented in this study.

## Conclusions

Atherogenesis involves the switching of gene expression towards pro-atherogenic genes in vascular cells. Multiple risk factors including oscillatory blood flow, hypertension, DM, smoking, alcoholism, sedentary lifestyle, obesity, high cholesterol, high triglycerides and other vascular stressors are involved in inducing pro-atherogenic pathological changes in vasculature.

At the core, it is the pathological alterations in vascular homeostasis that lead to atherogenesis and ultimately to the development of atherosclerosis. In this process of disease development, multiple factors play crucial roles. These factors include ageing, stem cell exhaustion and niche impairment, pathological alterations in vascular microenvironment, chronic inflammation and dysregulations in cell signaling pathways: Wnt, Notch, Hh pathway and others. This interconnected loop of pro-atherogenic vascular changes alters the gene expression of vascular cells.

Ageing impacts vascular homeostasis profoundly and in turn contributes to switching of vasculature towards atherogenesis. When the pathologic alterations in epigenetics, genetics, regulatory genes, microenvironment and vascular cell biology accumulate beyond a specific threshold, then the disease begins to express itself phenotypically. The pathological alterations are due to the shifts in ‘switches’ which maintain vascular homeostasis. This switching disrupts the physiologic gene expression of vascular cells and contributes to atherogenesis.

Atherosclerosis originates due to dysfunction in homeostasis of vascular cell types such as ECs, VSMCs, immune cells and fibroblasts. This onset is consequence of alterations that first begin with sensitive genes and accumulate over time, leading to cascade of alterations extending over wide range of genome. In this aspect, the role of proatherogenic epigenetic alterations induced by oscillatory blood flow and other risk factors are most significant. Such changes set in motion a cascade of disease unfolding mechanisms that result in endothelial dysfunction, dedifferentiation of VSMCs, alterations in intermediate filament content, accumulation of foam cells, fatty streak and chronic inflammation. There are cascades of pro-atherogenic changes in cell circuitry and gene expression, ranging from alteration in signaling pathways to alteration in transcription factors. Such changes play key roles in the development of atherosclerosis. In vasculature, this interconnected loop of pathologic changes in gene expression makes the microenvironment, cell signaling pathways and vascular maintenance mechanisms further dysregulated. The interactions and cross talk among pro-atherogenic alterations further fuel the disease development.

All the altered factors involved in pro-atherogenic vascular switching such as signaling pathways, gene expression, epigenetics, dysregulations in cell biology and others, they all contribute towards the decline in vascular homeostasis. The greater this decline and alterations are, the more advanced the atherogenesis becomes.

## Data Availability

The datasets supporting this article are included within the article, in the ‘References’ section.

## Abbreviations

Shh: Sonic hedgehog
TGF: Transforming growth factor
VEGF: Vascular endothelial growth factor
FGF: Fibroblast growth factor
IGF: Insulin-like growth factor
HGF: Hepatocyte growth factor
EGF: Epidermal growth factor
CREB: cAMP response element-binding protein
PTEN: Phosphatase and tensin homolog
ET – 1: Endothelin – 1
NF-κB: Nuclear factor kappa-light-chain-enhancer of activated B cells
TNF: Tumor necrosis factor
EGFR: Epidermal growth factor receptor
NGF: Nerve growth factor
AD: Alzheimer’s disease
BDNF: Brain-derived neurotrophic factor
GSK: Glycogen synthase kinase
C- Abl: C - abelson murine leukemia viral oncogene homolog
MAPK: Mitogen-activated protein kinase
IFN: Interferon
TFs: Transcription factors
NOs: Nitric oxide synthases
LDL: Low-density lipoprotein
BMP: Bone morphogenetic proteins
ROS: Reactive oxygen species
AGEs: Advanced glycation end products
CRP: C-reactive protein
ILs: Interleukins
ICAM: Intercellular adhesion molecule
ACE: Angiotensin-converting-enzyme
MMPs: Matrix metalloproteinases
MCP – 1: Monocyte chemoattractant protein-1
PGs: Prostaglandins
SDF 1: Stromal cell-derived factor 1
PDGF: Platelet-derived growth factor
EGF: Epidermal growth factor
ECM: Extracellular matrix
EPO: Erythropoietin
SFRP 2: Secreted frizzled related protein 2
DM: Diabetes mellitus
HDL: High-density lipoprotein
cAMP: Cyclic adenosine monophosphate
VSMCs: Vascular smooth muscle cells
ECs: Endothelial cells
HSV: Herpes simplex virus
CMV: Cytomegalovirus
PDGF: Platelet-derived growth factor
ECM: Extracellular matrix
JNKs: C-Jun N-terminal kinases
RA: Retinoic acid
Hh: Hedgehog
CVS: Cardiovascular system
CAM: Cell adhesion molecules
HIF 1: Hypoxia-inducible factors
TSP – 1: Thrombospondin 1
MAPK: Mitogen-activated protein kinase
